# HMPAS: Human Membrane Protein Analysis System

**DOI:** 10.1186/1477-5956-11-S1-S7

**Published:** 2013-11-07

**Authors:** Min-Sung Kim, Gwan-Su Yi

**Affiliations:** 1 Department of Information and Communications Engineering, KAIST, Daejeon 305-701, South Korea; 2 Department of Bio and Brain Engineering, KAIST, Daejeon 305-701, South Korea

## Abstract

**Background:**

Membrane proteins perform essential roles in diverse cellular functions and are regarded as major pharmaceutical targets. The significance of membrane proteins has led to the developing dozens of resources related with membrane proteins. However, most of these resources are built for specific well-known membrane protein groups, making it difficult to find common and specific features of various membrane protein groups.

**Methods:**

We collected human membrane proteins from the dispersed resources and predicted novel membrane protein candidates by using ortholog information and our membrane protein classifiers. The membrane proteins were classified according to the type of interaction with the membrane, subcellular localization, and molecular function. We also made new feature dataset to characterize the membrane proteins in various aspects including membrane protein topology, domain, biological process, disease, and drug. Moreover, protein structure and ICD-10-CM based integrated disease and drug information was newly included. To analyze the comprehensive information of membrane proteins, we implemented analysis tools to identify novel sequence and functional features of the classified membrane protein groups and to extract features from protein sequences.

**Results:**

We constructed HMPAS with 28,509 collected known membrane proteins and 8,076 newly predicted candidates. This system provides integrated information of human membrane proteins individually and in groups organized by 45 subcellular locations and 1,401 molecular functions. As a case study, we identified associations between the membrane proteins and diseases and present that membrane proteins are promising targets for diseases related with nervous system and circulatory system. A web-based interface of this system was constructed to facilitate researchers not only to retrieve organized information of individual proteins but also to use the tools to analyze the membrane proteins.

**Conclusions:**

HMPAS provides comprehensive information about human membrane proteins including specific features of certain membrane protein groups. In this system, user can acquire the information of individual proteins and specified groups focused on their conserved sequence features, involved cellular processes, and diseases. HMPAS may contribute as a valuable resource for the inference of novel cellular mechanisms and pharmaceutical targets associated with the human membrane proteins. HMPAS is freely available at http://fcode.kaist.ac.kr/hmpas.

## Background

Membrane proteins are proteins that act as an interface between the outside environment and the inside cellular processes. Therefore, they paly essential roles in various cellular functions, such as transporting molecules across membranes, sending and receiving chemical signals, anchoring other proteins at the membrane, and facilitating cell-cell communication [1]. They are also assumed to be major therapeutic targets. This is well supported by the fact that more than 60% of approved drug targets are localized in membrane [2].

Such biologically and therapeutically important membrane proteins are normally classified depend on how they locate in the membrane. The integral membrane protein (IMP) has peptide sequence region embedded in the membrane. In contrast, a lipid-anchored protein (LAP) is a protein attached to the lipid bilayer though a post-translationally attached lipid anchor rather than buried sequence regions in the membrane. Therefore, the two proteins cannot be separated without disrupting the membrane with detergent. The other is peripheral membrane protein (PMP), which is localized in the membrane by interacting with lipid head groups of the membrane or IMPs. Because of the significance of membrane proteins, there have been various efforts to construct membrane protein related resources. However, most of these efforts were concentrated on constructing databases for certain membrane protein group such as ion channel [3,4] and G-protein coupled receptor (GPCR) [5-8]. Although these databases provide a manually curated list of membrane proteins and their hierarchical classification information, they only cover small portion of entire membrane proteins. Therefore, it is difficult to infer specific characteristics of interesting protein groups by comparing with other membrane proteins that are scattered in different places. On the other hand, subcellular localization resources offer abundant amounts of proteins localized in various membrane regions, but they don't provide functional classification of these proteins. There is also a plant membrane protein database [9] which collects membrane proteins with *Arabidopsis thaliana *as a reference model. This database provides comprehensive information of plant membrane proteins including various sequence features. However, it doesn't provide classification of the collected proteins just like the subcellular localization resources. Membrane protein structure databases can be another source to retrieve membrane proteins [10], but they only contain a limited number of proteins that have experimentally validated structure information. This absence of comprehensive membrane protein database, which covers entire membrane proteins with their functional classification information, prevents the identification of both the common and specific characteristics of diverse membrane protein groups. This identification can be critical knowledge to predict novel proteins for a specific membrane protein family, to understand their mechanism of action, and to estimate novel uses of these proteins as drug targets.

In such circumstance, we proposed a comprehensive human membrane protein database in our previous study [11]. To construct this database, we collected human membrane proteins from various types of membrane protein related resources. Novel membrane protein candidates were also predicted by collecting membrane protein orthologs in other species and performing our novel membrane protein classifiers that can predict membrane proteins with their type of interaction with the membrane. Though these series of construction procedures, the database could provide the largest human membrane protein dataset compared to other resources. The collected membrane proteins were then grouped based on subcellular localization, molecular function, and type of interaction with the membrane.

In this research, we constructed a system to analyze the comprehensive information of human membrane proteins. For the construction of analysis system, the human membrane protein dataset was updated with the latest dataset collected from related resources. In addition to the updated human membrane proteins, we also constructed new feature information dataset for the membrane proteins. The number of integrated resources to construct the feature information was significantly increased including protein domain, pathway, disease, and drug. Furthermore, we integrated the disease and drug information by adapting a standardized disease classification system. This integration enables our system to retrieve all membrane proteins related with the target disease and to derive meaningful associations between diverse protein groups and diseases. The structure information of human membrane proteins was also newly added. After the construction of the comprehensive information of human membrane proteins, we implemented tools to analyze the comprehensive information. We built a feature enrichment tool to identify novel sequence and functional features of classified membrane protein groups. The sequence analysis tool was also implemented to extract various sequence features from protein sequences. We integrated 8 sequence prediction tools and our novel membrane protein classifiers to analyze protein sequences. Finally, we constructed a web interface of this system to support researchers to use the tools to analyze membrane proteins and to retrieve organized information of individual proteins.

## Methods

### Construction of human membrane protein dataset

**Figure 1:**
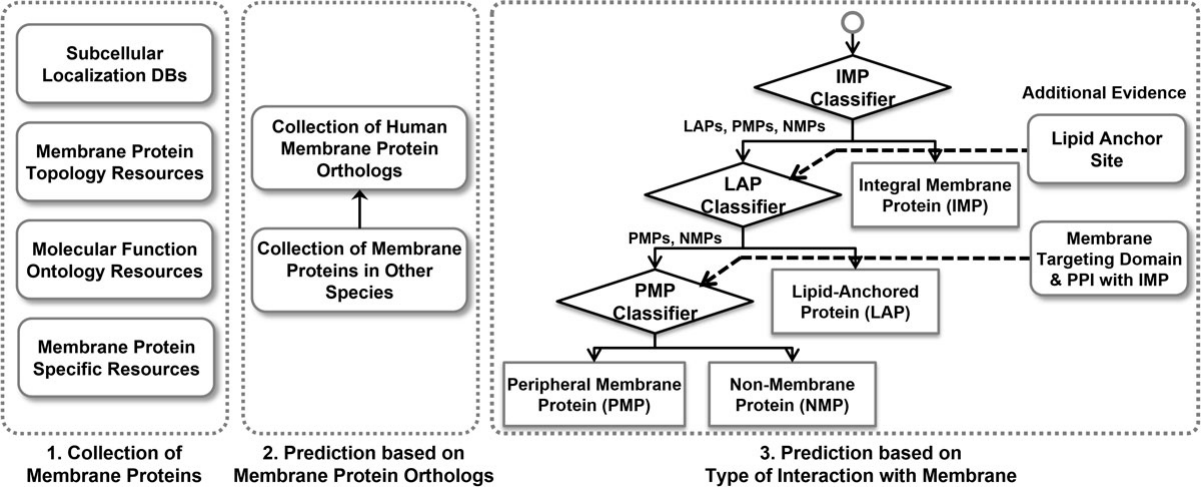
**Entire processes for the construction of human membrane protein dataset in the HMPAS**. The construction procedure was composed of 3 steps. Known membrane proteins were collected from 4 different types of resources. Novel membrane proteins were predicted by searching membrane protein orthologs in other species. The collected proteins were classified based on their type of interaction with the membrane. After the classification, the classified proteins were used to train the classifiers for each type of interaction with the membrane and were then applied to predict the novel membrane proteins.

For the construction of human membrane protein analysis system, we generated human membrane protein dataset as we did for the construction of membrane protein database [11]. This dataset is comprised of collected membrane proteins from diverse resources and predicted membrane proteins by searching homologous membrane proteins in other organisms and by performing our membrane protein classifiers, as depicted in Figure 1. The known human membrane proteins were gathered from 4 different types of resources. The subcellular localization resource is a representative resource that provides proteins localized in various membrane regions. The membrane localized proteins were collected from 8 subcellular localization resources; UniProt Subcellular Locations (SL) [12], UniProt Keywords, GO Cellular Component (CC) [13], DBSubLoc [14], eSLDB [15], Organelle DB [16], LOCATE [17], and DMBLoc [18]. Membrane protein topology resource provides transmembrane proteins with their embedded sequence regions. These proteins were gathered from 3 membrane protein topology resources; UniProt sequence section, TOPDB [19], ExTopoDB [20]. In molecular function ontologies, there were some terms that is highly correlated with membrane proteins such as "KW-0407 Ionic channel" and "GO:0004888 transmembrane signaling receptor activity". The proteins annotated with such terms were collected from UniProt Keywords and Gene Ontology (GO) Molecular Function. As a last step, we retrieved membrane proteins from 7 well-known membrane protein group databases; GPCRDB, gpDB, 7-transmembrane G-linked receptors, VKCDB, TCDB [21], IUPHAR-DB, and KEGG BRITE [22]. Each collected protein was assigned with a unique UniProt accession ID so that it could be distinguished from the other proteins. It was also allocated with evidence codes by taking into consideration of its original evidence codes available from sources. The collected protein dataset has some redundancy in terms of identical sequences and sub-fragments. The redundant sequences can make user confuse to search appropriate proteins and make bias when the collected proteins are used as training set for further researches. UniRef100 [23] dataset was used to remove the redundancy because it provides clustered group of such redundant sequences. It also provides a representative protein of each group by considering information contents of member proteins.

Novel human membrane proteins can be predicted by searching the membrane protein orthologs in other organisms. Among various eukaryotic organisms, 55 model organisms, which are currently available in Ensembl database [24], were selected. The collection procedures for the human membrane proteins were identically performed for the membrane proteins in other species. Before searching the orthologs, only membrane proteins annotated with reliable evidence code were used. Novel human membrane protein orthologs were predicted by mapping the membrane proteins in other species to human orthologs based on orthologous relationships provided by Ensembl Compara. Membrane proteins that had already been collected were discarded from the predicted dataset.

Membrane proteins can be broadly classified into 3 distinct classes based on how they locate in the membrane. Therefore, we classified the collected proteins to reflect the different natures of membrane proteins before the prediction. After the classification, we implemented a random forest classifier for each type of membrane protein. Most IMPs have sequence regions that are assumed as hydrophobic because they exist in the hydrophobic inner layers of membrane. The hydrophobic region can be a distinctive feature compared to other proteins. However, PMPs don't have such common properties related with the localization in the membrane. This makes it difficult to distinguish PMPs from various non-membrane proteins. LAPs are similar to PMPs but comprise relatively well-known membrane protein groups such as G proteins. Therefore, we arranged the classifiers in sequential order. After the arrangement, additional evidence information for the PMP and LAP classifiers were integrated to increase the overall confidence of the predicted membrane proteins. For the LAP classifier, existence of lipid-anchor sites was further checked. Currently known lipid-anchor sites from dbPTM [25] and predicted sites from related prediction tools were used; Myristoylator [26] and FragAnchor [27]. Known membrane protein targeting domains and existence of interaction relationship with IMPs were also checked for the PMP classifier. Nine representative membrane targeting domains were retrieved from MeTaDor [28]. The protein-protein interaction information stored in our comprehensive protein interaction database [29] was used to search the interaction relationships.

### Classification of membrane proteins

Although we gathered human membrane proteins from scattered resources, it is complicated to extract meaningful information from such collection of various protein groups. To deduce common and specific characteristics features from the membrane protein dataset, they have to be hierarchically classified into smaller groups that share common characteristics. For this classification, we classified membrane proteins based on type of interaction with the membrane, subcellular localization, and molecular function. The detail procedure of this classification was explained in our previous research [11]. At first, the membrane proteins were classified into IMPs, PMPs, and LAPs. The collected membrane proteins were also categorized based on what kinds of membrane they interact with. Major categories of this localization based classification are plasma membrane and organelle membrane. The major classes are further classified with additional 43 sub-classes. Molecular function based classification is the last categorization for the membrane proteins. This function based classification is integration of different classification structures from membrane protein specific databases and molecular function ontologies. The root category terms are "Transporter", "Receptor", "Enzyme", and "Others". The child classes of "Others" are "Structural molecule", "Cell adhesion molecule", and "Ligand". Current molecular function based classification is composed of 1,401 hierarchical classes.

### Characterization of membrane proteins with sequence features

We characterized the collected membrane proteins with three different sequence features; membrane protein topology, lipid-anchor site, and domain. For transmembrane proteins, it is important to know which sequence regions of the proteins in the membrane and which sequence regions are outside of the membrane. This information can be assumed as a low resolution structure of each transmembrane protein. In recent years, this topology information is also frequently used to identify linear motifs conserved in the transmembrane regions, which can be valuable constraints for protein structure modeling. The PDBTM and UniProt sequence annotation sections were used gather known topology region information. We also integrated and performed 5 available membrane protein topology prediction tools to unveil the topology information of unknown transmembrane proteins: TMHMM [30], S-TMHMM [31], SCAMPI [32], HMMTOP [33], and PHOBIUS [34].

Lipid-anchor may attach a protein to the lipid bilayer of a membrane. It is a distinctive feature of lipid-anchored proteins compared to other membrane proteins. Known lipid anchor site information was gathered from dbPTM database. We also collected predicted lipid-anchor sites from 2 available prediction tools: Myristoylator and FragAnchor.

Protein domain is a conserved part of a protein sequence which is assumed as a functional or structural unit of protein [35,36]. It is usually associated with interacting with other molecules or performing certain biological functions. We integrated the domain information of membrane proteins from 6 resources; InterPro [37], Pfam [38,39], PROSITE [40], PRINTS [41], GENE3D [42], and SUPERFAMILY [43].

### Characterization of membrane proteins with functional features

The molecular function classification of a membrane protein depicts functional abilities of the protein itself. In contrast, biological process is a cellular activity that is organized with series of molecular functions or events. Therefore, this information can explain functional roles of membrane proteins by interacting with other molecules. UniProt Keywords and GO Biological Process were used to agglomerate the biological process information of membrane proteins. Although functional coverage of the biological process encompasses signaling and metabolic processes, annotated member proteins and detail description of cellular mechanism can be limited compared to pathway information. The pathway can also be used to describe underlying mechanism of various disease pathologies. Therefore, we constructed comprehensive pathway information for membrane proteins. For the construction, we integrated 8 pathway resources for this analysis system; KEGG, NCI PID [44], PharmGKB [45], Reactome [46], NETPATH [47], PANTHER Pathway [48], UniPathway [49], and BioCarta.

Pharmaceutical information was gathered to characterize phenotypic effects of membrane proteins beyond cellular space and to increase the significance of this system for pharmaceutical research. We collected known membrane protein targeting drugs and disease associated membrane proteins and integrated them based on International Classification of Diseases-10th Revision-Clinical Modification (ICD-10-CM) classification system. Disease association information of membrane proteins was collected from PharmGKB, OMIM [50], KEGG DISEASE, Genetic Association Database [51], and Cancer Gene Census [52]. For the collection of membrane protein targeting drugs, we aggregated the information from Drugbank [53], KEGG DRUG, and TTD [54]. Although this collection of information is meaningful to reveal pharmaceutical importance of individual membrane protein, it is difficult to infer associations between classified membrane protein groups and the pharmaceutical information. Type 2 diabetes mellitus, for instance, is stored with different names in the genetic disease association databases: "DIABETES MELLITUS, NONINSULIN-DEPENDENT; NIDDM" (OMIM), "Type II diabetes mellitus" (KEGG DISEASE), "diabetes, type 2" (Genetic Association Database), and "Diabetes Mellitus, Type 2" (PharmGKB). Furthermore, the target disease information of drug is written with sentences in drug indication field. In addition to these heterogeneous representations, there are no hierarchical relationships between these disease terms in the collected resources. If a researcher wants to retrieve diabetes mellitus associated membrane proteins, the proteins from child terms, which are composed of type 1 diabetes and type 2 diabetes, have to be retrieved in addition to the proteins annotated with the diabetes mellitus term. Because of these problems, it is complicated to retrieve all membrane proteins related with target disease and to deduce meaningful associations between protein groups and diseases. Therefore, the collected information needs to be integrated by using a standardized disease classification system. For the integration, we firstly retrieved disease names from disease databases and drug indication fields from drug databases. The Unified Medical Language System (UMLS) terms were extracted from the text set by using MetaMap [55]. Because the UMLS was intended to be made to support various types of biomedical terms, the mapping results contain various types of terms in addition to disease terms. Therefore, we additionally selected a standardized disease term set; ICD-10-CM (International Classification of Diseases, 10th Revision, Clinical Modification). We converted the various types of UMLS IDs into ICD-10CM IDs by using mapping information provided by UMLS Metathesaurus [56]. As a result, the independent disease and drug information were integrated according to the ICD-10-CM disease classification hierarchy.

In addition to the disease classification, there are drug classification codes which classify drugs based on their therapeutic characteristics. Therefore, we additionally grouped collected drugs based on their therapeutic classes. The Anatomical Therapeutic Chemical (ATC) classification system was used because it is a drug classification code that is managed by WHO. We retrieved drug-ATC code mapping information from integrated drug databases and mapped each drug to the ATC hierarchy.

### Characterization of membrane protein with structure feature

Although structure information of membrane proteins is one of major features to understand mechanisms of action and to design how to use them in various applications, current number of membrane proteins with experimentally validated structure is limited because the lipids surrounding the proteins in membranes interfere with generally used experimental techniques [57]. In this circumstance, the known structure information can be valuable asset that can be used for computational structure modeling of unknown membrane proteins. Therefore, we integrated currently known structure information of membrane proteins by collecting PDB IDs from PDBTM and UniProt.

### Identification of novel features from membrane protein groups

The collected membrane proteins were classified into smaller groups. The classified proteins were further characterized with various sequence and functional features in this database. Because of the integration of such comprehensive information in one place, we could identify the specific features of each membrane protein group. The identified features can reveal novel associations between proteins groups and features. To measure the specificity of a feature in each protein group by comparing with other proteins, we constructed a functional enrichment tool which is a commonly used method for the interpretation of functional roles of certain protein group. The enrichment analysis was performed for each protein group and identified features were integrated into this system. The enrichment procedure was implemented by referencing our previous functional module enrichment analyses [58,59]. The significance was evaluated by using hypergeometric test.

### Identification of features from protein sequence

In our previous research related with the membrane protein database, there was no method to support analyzing user's input sequence. To identify various features from the input sequence, we integrated 8 prediction tools and our membrane protein classifiers. This tool performs three different analyses at once. Homologs of the input sequence among the membrane proteins of HMPAS were searched using BLAST [60]. The sequence prediction tools, which were used for the characterization of unknown human membrane proteins, were also integrated to identify sequence features from the input sequence. In addition to searched proteins in the alignment result, the identified features can also be used as a query to search related membrane proteins. Among the sequence features, predicted membrane protein topology and matched domains were visualized on the query sequence. The visualization module used Scalable Vector Graphics (SVG) to generate the images. Finally, the membrane protein prediction is performed on the input sequence. The prediction is carried out with the same prediction procedure that was used to predict novel human membrane protein candidates according to their type of interaction with the membrane.

## Results

### Current statistics of human membrane protein dataset

The current number of membrane proteins, which was recently updated, is summarized in Table 1. We gathered 28,509 known membrane proteins from the integrated resources. Among the predicted membrane protein candidates, 345 proteins were predicted by searching membrane protein orthologs in 55 other species. A total of 7,731 novel membrane protein candidates were also predicted by using the 3 distinct membrane protein classifiers, which considered their type of interaction with the membrane.

**Table 1:** Current membrane protein dataset in HMPAS

Resource Type	Protein Number
Collected human membrane proteins	28,509
Predicted membrane proteins from membrane protein orthologs in 55 other organisms	345
Predicted membrane proteins from membrane protein classifiers	7,731

Total human membrane proteins	36,585

### Pharmaceutical features of membrane proteins

Membrane proteins are considered as major pharmaceutical targets. Therefore, among the various sequence and functional features, we investigated pharmaceutical features of membrane proteins as a case study. For the analysis, we measured the coverage of membrane proteins by current drug targets and investigated specific features of membrane proteins in terms of pharmaceutical information. Among currently known proteins targeted by FDA approved drugs from TTD and DrugBank, about 69.0% of proteins were membrane proteins. If experimental drugs are also considered, 65.1% of the target proteins were included in the dataset. This suggests the usefulness of targeting membrane proteins compared to proteins localized in other cellular compartments.

**Figure 2:**
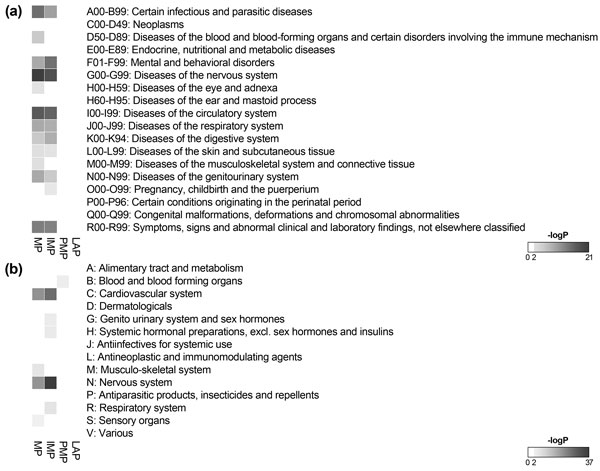
**Heatmap representing the degree of associations between disease/therapeutic classes and membrane proteins**. The disease associations were measured with first level classes of the ICD-10-CM, which were used to integrate the disease and drug information (a). For the therapeutic associations, the first level codes of ATC, which were used for the classification of integrated drugs depend on their therapeutic characteristics, were used (b). The degree of association was measured by hypergeometric test with FDR multiple testing correction. Only significantly enriched results with p-value below 0.01 are colored in the diagram. MP means all membrane proteins in this database.

In addition to the coverage of membrane proteins, we also analyzed associations between the collected membrane proteins and disease/therapeutic classes. The disease and drug information was integrated based on ICD-10-CM classification system. To analyze overall tendencies of disease associations, we selected chapter terms, which are 1^st ^level classes in ICD-10-CM hierarchy, of the ICD-10-CM. As illustrated in Figure 2, membrane proteins were closely involved in infectious and parasitic diseases, mental and behavioral disorders, diseases of the nervous system, and disease of circulatory system. The diseases association is also similarly shown for IMP. The PMP and LAP have no significant associations with the disease classes. In the therapeutic association aspects, we selected first level of ATC codes and used them for further analysis. Membrane proteins were highly targeted by drugs correlated with the nervous system and cardiovascular system. The both results indicated that membrane proteins were promising targets for diseases associated with the nervous and circulatory system.

### Web interface

**Figure 3:**
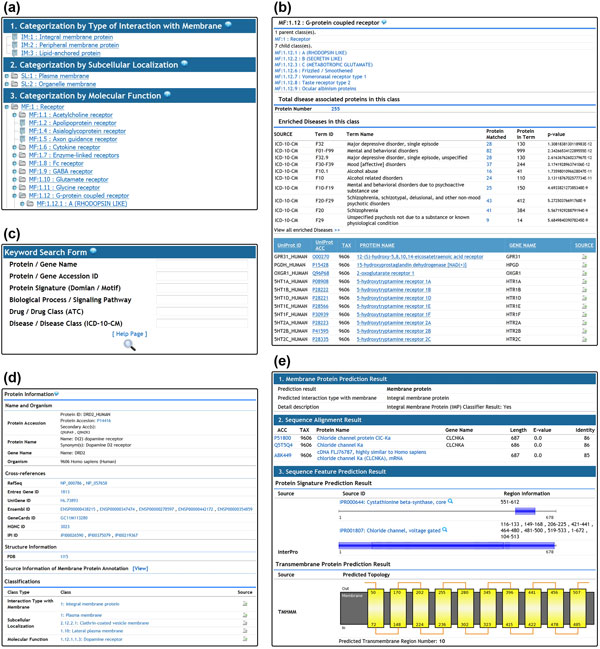
**Screenshot of the HMPAS showing its main web interfaces**. Users can browse the hierarchical structure of membrane protein classes and click to view detailed information of the target class (a). The class information page shows its direct parent and child terms and summarized view of the identified characteristic features of member proteins (b). The HMPAS permits searching via keywords with 6 different fields (c). The information page of the target protein provides its general information, classification annotation, and sequence and functional features in an organized format (d). It also provides a sequence analysis page for searching homologous proteins in the system, extracting sequence features in the query sequence, and predicting novel membrane protein (e).

The HMPAS is accessible at http://fcode.kaist.ac.kr/hmpas. The data contents of HMPAS are stored in an Oracle (http://www.oracle.com/) relational database. The web service was developed with JavaServer Pages and JavaScripts based on Tomcat servlet container (http://tomcat.apache.org/). The DHTML extensions Tree library was used to dynamically load the hierarchical tree of the classifications. The main interface of the HMPAS is composed of browsing of the classified membrane proteins, browsing of the membrane proteins with their features, searching via keywords, and analysis of sequence, as shown in Figure 3.

The HMPAS supports browsing the hierarchical structure of membrane protein classes. The browser page is divided into 3 parts according to the classification types, and users can easily explore the membrane proteins under specific categories in the hierarchical structure. Each class is linked to a detailed information page for the class. The class page shows the direct parent and child classes in the hierarchies, allowing the user to move up and down without loading all classes. Each class page also presents the specific sequence and functional features identified by the enrichment tool. The identified features were categorized based on its feature type and sorted with their p-value.

Users can also browse the membrane proteins of the HMPAS with their annotated features rather than the classification hierarchies. If the user sets the feature type and target resource in the drop-down menu, the annotated features of membrane proteins are listed, and each annotation term is linked to its member protein page.

Users can search against the HMPAS by typing name, accession ID, protein signatures, biological processes, targeting drugs, and diseases. The search is performed by typing keywords in any field separately or in several fields simultaneously. The search result shows the list of matched membrane proteins, and each protein is linked to a detailed protein information page. The protein information page shows all available characteristic features of corresponding proteins and cross-reference links to several external databases. Each annotated feature in the protein information page can also be used to search for other proteins that have the same feature, by clicking the search icon next to the feature. Users can also retrieve the integrated source information which is reason for collecting the protein as a membrane protein and allocating the protein with current class annotation.

In the sequence analysis menu, users can analyze the membrane protein characteristics of their input sequence by using the sequence analysis tool. The sequence alignment option can be modified with E-value and identity. The analysis result contains homologous membrane proteins in HMPAS, sequence features identified in the input sequence, and membrane protein prediction result. The proteins in the alignment result and predicted sequence features are linked to the membrane protein information page.

## Conclusions

In this study, we constructed a system that integrates comprehensive information of human membrane proteins and analysis tools to examine the comprehensive information. The HMPAS collects membrane proteins from various resources that are scattered in different locations and provides novel membrane protein candidates predicted by using membrane protein orthologs and our membrane protein classifiers that can predict membrane proteins with their type of interaction with the membrane. In comparison with other IMP databases, the HMPAS additionally covers the information of biologically important LAPs and PMPs. This comprehensive collection of membrane proteins can be further used to analyze regulatory networks of membrane proteins [61]. Moreover, it supports hierarchical function classification information of collected membrane proteins compared to subcellular localization resources.

The constructed membrane protein analysis tools provide ways to analyze numerous features of the membrane groups and input protein sequences. The collected membrane proteins were classified based on three different types of aspects. Our enrichment tool was used to identify novel sequence and functional features of the classified membrane proteins. The analysis results are available through our web interface and enable researchers obtain information on which membrane protein group can be effectively used for therapeutic purposes and can examine which sequence and functional features such proteins have. Users can also characterize their input sequences by retrieving information of homologous proteins or identifying various sequence features.

Therefore, the HMPAS will be a valuable resource for the research of cellular functions of membrane proteins by revealing their novel features related with their cellular mechanisms and the identification of novel drug targets by supporting with comprehensively integrated pharmaceutical information of membrane proteins.

## Competing interests

The authors declare that they have no competing interests.

## Authors' contributions

MK integrated membrane protein dataset, analyzed the dataset, and constructed web-based system. GSY conceived and supervised this study. MK and GSY wrote the manuscript. All authors read and approved the final manuscript.

## Declarations

The publication costs for this article were funded by the corresponding author.

This article has been published as part of *Proteome Science *Volume 11 Supplement 1, 2013: Selected articles from the IEEE International Conference on Bioinformatics and Biomedicine 2012: Proteome Science. The full contents of the supplement are available online at http://www.proteomesci.com/supplements/11/S1.
